# Hybrid Ensemble Model for Knee Osteoarthritis Grading: Integrating CNNs with GLCM Features and XAI

**DOI:** 10.3390/diagnostics16040539

**Published:** 2026-02-11

**Authors:** Lubna Mohammad Almusa, Turky Nayef Alotaiby, Hanan Saeed Murayshid, Rawad Awad Alqahtani

**Affiliations:** 1 Department of Artificial Intelligence Sciences, College of Computer and Information Sciences, Princess Noura Bint Abdulrhman University, Riyadh 11564, Saudi Arabia; 2 Artificial Intelligence and Robotics Institute, King Abdulaziz City for Science and Technology (KACST), Riyadh 12345, Saudi Arabiahmurayshed@kacst.gov.sa (H.S.M.)

**Keywords:** knee osteoarthritis, Kellgren–Lawrence grading, X-ray radiographs, deep learning, ensemble learning

## Abstract

**Background**: Knee osteoarthritis (KOA) is characterized by cartilage degradation and joint-space narrowing, resulting in increased friction and observable structural damage. **Methods**: This study introduces a composite hybrid framework for the automatic classification of KOA severity using anteroposterior knee X-ray images. The methodology applies joint-centered cropping and data augmentation to standardize inputs and uses class weighting to mitigate class imbalance. Deep features extracted from fine-tuned ResNet-101 and EfficientNetB7 models are integrated with handcrafted Gray Level Co-occurrence Matrix (GLCM) texture descriptors, and the final predictions are obtained using a soft-voting ensemble. **Results**: the proposed ensemble achieves 73% test accuracy (macro-F1 ≈ 0.70; weighted-F1 ≈ 0.73) in a four-class setting (KL-0, KL-2, KL-3, and KL-4). Additional experiments across different classification setups demonstrate consistent performance trends, while Grad-CAM indicates that the model primarily focuses on the joint region. Overall, **Conclusions**: combining ensemble deep learning with complementary handcrafted texture features provides a reliable and interpretable approach for grading radiographic KOA severity.

## 1. Introduction

**Figure 1:**
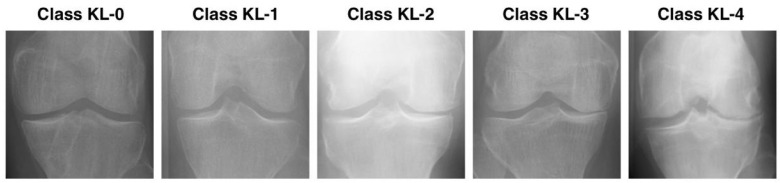
Kellgren–Lawrence grading criteria for knee osteoarthritis (0–4).

Knee Osteoarthritis (KOA) is the most common form of osteoarthritis, representing the degeneration of one’s skeletal joints by the gradual loss of cartilage in the knee joint, causing pain, stiffness, and impaired mobility, as well as functional impairment [1]. KOA is particularly common among elderly individuals and greatly impairs their quality of life. The effective management of this condition depends on early diagnosis, but traditional diagnostic methods, such as manual evaluation of radiographic images, are subjective and time-consuming [2]. In the past few years, deep learning (DL) has shown great promise in automating KOA assessment through analysis of knee X-ray images [3,4]. One of the most widely used standards for grading KOA severity is the Kellgren-Lawrence (KL) grading system [5,6]. The KL grading system assigns a KOA grade from 0 to 4. Grade 0 is a normal joint with no OA signs radiographically; grade 1 is the doubtful/unsure changes; grade 2 is the minimal degeneration; grade 3 is the moderate structural damage; and grade 4 is the heavy joint destruction with a much-lowered joint space. The complete criteria of the KL grading are presented in Figure 1.

Although deep learning has made great achievements, there are also three key issues still existing in clinical practice: (i) label ambiguity, particularly for KL-1 cases, which are difficult to distinguish from adjacent grades [7], KL-1 represents a borderline radiographic stage that typically shows minimal osteophytic changes without definitive joint-space narrowing, which can lead to subjective classification. This ambiguity is especially pronounced because KL-1 lies between healthy joints (KL-0) and confirmed KOA (KL-2) and is often described as “doubtful” KOA [8]. Accordingly, prior studies have reported substantial inter-observer variability in grading, particularly for early-stage cases [9,10]. (ii) imbalanced data, where the normal cases are often overrepresented compared to moderate and severe cases; and (iii) the ongoing need for interpretable and explainable predictions that align closely with clinical reasoning and support decision-making in real-world settings.

This study focuses on multiple KL grading schemes, including four-class KL-(0, 2, 3, 4) and five-class KL-(0, 1, 2, 3, 4) settings, to emphasize the ambiguity associated with KL-1 grades, as well as the three-class KL-(2, 3, 4) and binary classification between early KL-(0 + 1) and later-stage KL-(2 + 3 + 4) comparisons. The objective is to develop a light and efficient pipeline that (a) view-centers the tibio-femoral joint, (b) applies clinically meaningful data augmentation, and (c) employs class-weighted optimization to tackle dataset imbalance. Based on two state-of-the-art ImageNet-pretrained models—ResNet-101 and EfficientNet-B7—we propose a mixed feature extractor that combines global average-pooled CNN features with a six-dimensional Gray-Level Co-occurrence Matrix (GLCM)-based texture descriptor to incorporate handcrafted radiographic features at low overhead and enhance representational richness. In addition, we use a soft voting ensemble over the two model backbones to improve prediction stability. We hypothesize that the KL-1 grade introduces labeling ambiguity, negatively affecting grading reliability, and that a joint-focused, lightweight, deep-texture ensemble improves robustness and accuracy across grading settings.

This work makes the following key contributions:

1. We design and evaluate a multi-class and binary-class KL-grading configuration with joint-centrical cropping at a resolution of 104 × 224 to focus on the most informative knee regions.
2. We develop an enhanced preprocessing pipeline that integrates joint-centered cropping alongside histogram, contrast enhancement, normalization, denoising, CLAHE, and edge detection to improve X-ray quality and emphasize key knee joint structures for accurate classification.
3. We propose a hybrid deep-texture classifier that fuses CNN features with a lightweight GLCM prior to capturing complementary structural features.
4. We employ class-weighted training with a two-model ensemble (EfficientNet-B7 + ResNet-101), yielding improved robustness and performance across grading tasks.

The rest of the paper is organized as follows: Section 2 reviews related work in KOA classification using deep learning. Section 3 presents the proposed methodology, including the model architecture, preprocessing, and training strategy. Section 4 discusses experimental results and evaluation metrics. Finally, Section 5 concludes the paper with a summary of findings and directions for future research.

## 2. Related Work

Deep learning has achieved remarkable utilization in the medical imaging field [11], where it has been widely applied across various modalities, including X-ray, MRI, and ultrasound, for diagnostic classification tasks [12]. In the context of knee osteoarthritis (KOA), several works have explored CNN-based architecture, with ResNet being among the most frequently used for radiograph classification [13]. Further approaches have also been explored for the automatic detection and grading of KOA, demonstrating the broad applicability of deep learning techniques in this field [14,15,16].

Among these advances, the preprocessing pipeline has played a critical role in improving performance. The study in [5] implemented a preprocessing pipeline that cropped the knee joint region from X-ray images before training, removing irrelevant areas. The authors evaluated six pre-trained deep neural networks, including VGG16, VGG19, ResNet101, MobileNetV2, InceptionResNetV2, and DenseNet121, using data from the Osteoarthritis Initiative (OAI). They structured their experiments into binary and multi-class settings: binary classification to distinguish healthy from diseased knees and multi-class classification to grade severity. Among the models, ResNet101 achieved the best results across the different setups, highlighting the importance of both preprocessing and model selection in KOA severity prediction. At the same time, other studies relied on edge detection to highlight joint-space boundaries, such as [17]. Although CNNs perform automatic feature extraction, a limited number of studies have combined handcrafted features to strengthen deep representations, such as utilizing Gray-Level Co-occurrence Matrix (GLCM) features in diabetic retinopathy classification [18] or combining GLCM with LBP for KOA prediction [19], both of which yielded improved results.

Another line of research has focused on ensemble learning, which has consistently demonstrated improved robustness and performance compared to single-model baselines [20,21,22]. Despite the strong performance of deep learning, recent studies have continued to compare different paradigms, including traditional machine learning, CNN-based models, and more recent transformer architectures [23]. Notably, transformer-based solutions, including the Vision Transformer for KOA severity grading, the Selective Shuffled Position Embedding with Key-Patch Exchange [7], and multimodal frameworks like CLIP-KOA with symmetry-aware loss functions [24], have provided new perspectives for KOA classification.

At the same time, interpretability remains an important consideration; a systematic review emphasized the need for explainable AI in KOA diagnostics [25]. The seminal work in [26] introduced Grad-CAM as a technique to generate visual explanations for CNNs. In contrast, subsequent KOA-focused studies, such as [27], applied this method to highlight clinically relevant regions, confirming that models often attend to the joint space and subchondral margins. The authors in [28] presented an integrated framework of deep learning and explainable AI (XAI) methodologies to categorize the severity of knee osteoarthritis (KOA) from X-ray images through KL grading. They consider the multi-class and binary-class classification by fine-tuning various pre-trained CNN models, namely VGG, ResNet, and EfficientNetB7. For better interpretation, they used Grad-CAM visualizations, which revealed that the models tend to pay attention to clinically significant areas, such as the knee joint space. Interestingly, the binary classification simulations showed higher accuracy when the class variance was larger, especially in distinguishing between normal and severe KOA. However, the models showed limited performance when faced with ambiguous grades such as KL-1, which, as we discussed, reveals the difficulty of fine-grained grading of KOA.

While transformer-based architectures, including Vision Transformers [7,23] and multimodal frameworks like CLIP-KOA [1,24] have recently emerged as promising alternatives, CNN-based approaches continue to demonstrate competitive performance with practical advantages. Specifically, established CNN architectures like ResNet and EfficientNet benefit from mature ImageNet pretraining that transfers effectively to medical imaging tasks with moderate-sized datasets, offer superior computational efficiency, which is crucial for clinical deployment, and integrate seamlessly with handcrafted features to provide complementary representations. Recent comparative studies [5,22] confirm that well-optimized CNN frameworks achieve state-of-the-art results on KOA grading while maintaining lower computational overhead than transformer variants. Moreover, the explicit fusion of CNN features with texture descriptors, such as GLCM, provides interpretable radiographic characteristics that align with clinical assessment practices that enhance both performance and explainability. Across these varied avenues—spanning preprocessing pipelines, selective handcrafted feature integration, ensemble learning, and explainable AI—the open question is: can a hybrid deep learning framework that unifies these techniques achieve greater robustness and clinical reliability than existing approaches?

## 3. Methodology

**Figure 2:**
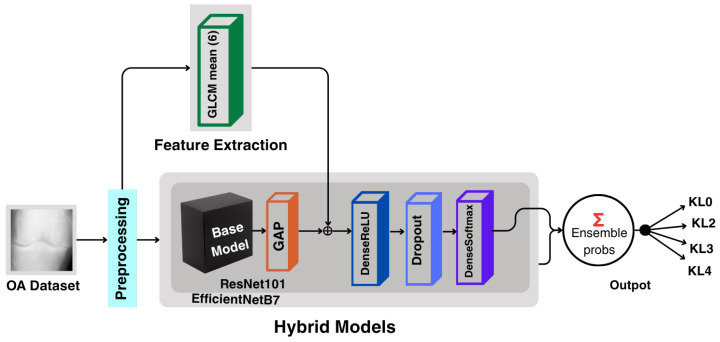
Methodological pipeline of the proposed approach. The framework includes preprocessing, feature extraction using deep models (ResNet101 and EfficientNetB7) and GLCM texture descriptors, followed by a soft-voting ensemble. GAP denotes Global Average Pooling, and KL0–KL4 represent Kellgren–Lawrence grades 0–4 for KOA severity.

The proposed pipeline commences with basic preprocessing and data augmentation on the X-ray images, followed by the extraction of handcrafted features using the Gray Level Co-occurrence Matrix (GLCM). These features are fused with learned representations from pre-trained CNN models, namely ResNet101 and EfficientNetB7. Both models pass through the training pipeline, and, finally, an ensemble strategy combines their outputs to produce the final classification for KOA, as illustrated in Figure 2.

In this study, two pre-trained convolutional neural network (CNN) architectures were employed as base models: ResNet-101 and EfficientNetB7.

### 3.1. Dataset

**Figure 3:**
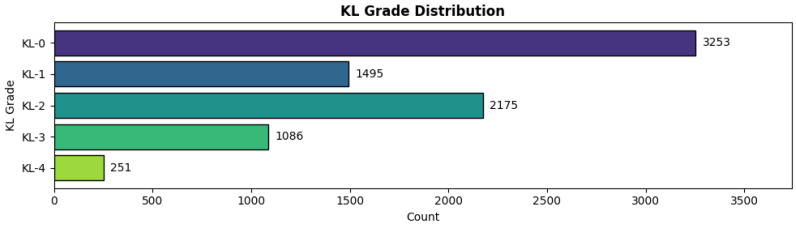
Class distribution across Kellgren–Lawrence (KL) grades (0–4) for the training, validation, and test splits.

In our experiment, we used the Knee Osteoarthritis Severity Grading Dataset [29], in which X-ray images were semi-automatically annotated using the Kellgren–Lawrence (KL) grading system (0–4). Developed at the University of Florida, the dataset comprised 8260 images, split into 70% (5778) for training, 10% (826) for validation, and 20% (1656) for testing. Figure 3 reports the class distribution across KL grades in each split, highlighting the imbalanced nature of the dataset, particularly for KL-4.

**Figure 4:**
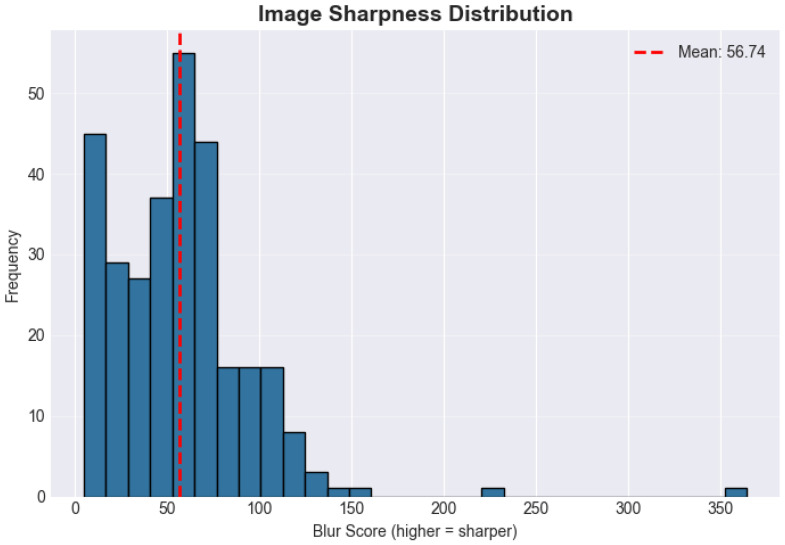
Distribution of image sharpness (blur) scores across the dataset (higher scores indicate sharper images).

Figure 4 shows the distribution of image sharpness scores across the dataset (higher is sharper).

### 3.2. Data Preprocessing

**Figure 5:**
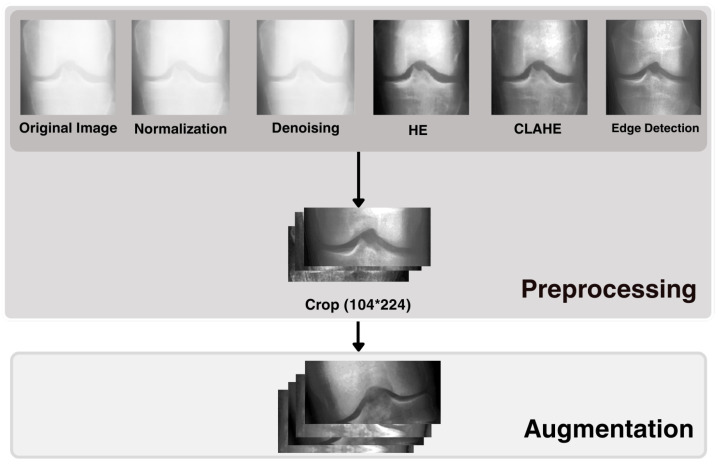
Preprocessing operations applied to knee X-ray images.

The preprocessing pipeline was intentionally designed as a sequence of complementary enhancement steps, rather than a single transformation, to incrementally refine the visual quality of knee X-ray images before feature extraction. The process began with (1) normalization to reduce intensity differences among images obtained under varying exposure settings. Next, (2) denoising was applied to attenuate high-frequency noise that frequently occurs in radiographs, thereby improving signal uniformity. This was followed by (3) histogram-based enhancement and (4) contrast-limited adaptive histogram equalization (CLAHE), which were used to locally increase contrast—especially in low-contrast regions around the knee joint—so that subtle structural details became more visible. (5) Edge detection was then employed as the final enhancement step to highlight intensity transitions and structural (anatomical) boundaries, which are important for recognizing joint-space narrowing and bony contours. As shown in Figure 5, these combined operations enhance the visual clarity of the joint area without modifying its underlying anatomical structure. By using an integrated series of enhancement methods rather than a single technique, the pipeline is designed to generate visually cleaner, structurally accentuated inputs, thereby supporting more robust feature learning in both CNN-based and texture-based models. All preprocessing procedures were implemented in MATLAB (https://www.mathworks.com/help/matlab/release-notes.html MATLAB R2024a, MathWorks, accessed on 29 January 2025).

Following the enhancement steps, the images were cropped to a fixed dimension of 104 × 224 pixels. Because the radiographs in this dataset are uniformly aligned, we removed 60 pixels from both the top and bottom of each image, preserving the central knee joint area while reducing irrelevant background. The resulting ROI was then resized to 104 × 224 pixels to ensure a consistent input format across all models, using the same cropping procedure as in [5].

To address class imbalance and enhance model generalization, each class was oversampled up to 1500 images and subjected to mild data augmentation. Given the sensitivity of medical images, extensive transformations risk altering clinically important structures; therefore, only limited and realistic augmentations were employed to avoid anatomically implausible changes. Vertical flips were omitted because they do not correspond to a valid orientation for knee radiographs, whereas horizontal flips were deemed acceptable. The complete set of applied transformations is listed in Table 1.

### 3.3. Deep CNN Backbones

Residual Networks (ResNet) were originally introduced by He et al. [30] to address the vanishing gradient problem in very deep architectures by using skip connections, which allow gradients to flow more effectively during backpropagation. EfficientNet, proposed by Tan and Le [31], introduced compound scaling of network depth, width, and resolution, achieving state-of-the-art accuracy with optimized computational cost.

In this study, we employed ResNet-101 (a residual network with 101 layers) and EfficientNetB7 (the largest model in the EfficientNet series) as our backbone architectures. Both networks were initialized with ImageNet-pretrained weights, and we fine-tuned them for the knee X-ray classification task by unfreezing the final 20 layers.

### 3.4. Handcrafted Feature Extraction

While deep CNN models can automatically learn abstract representations from images, complementary handcrafted features were also extracted to enrich the model with explicit texture descriptors. Specifically, the Gray Level Co-occurrence Matrix (GLCM) [32] was employed to capture second-order statistical properties of the knee joint region. For each image, GLCMs were computed at multiple pixel distances (d = 1, 2, 3) and orientations (0°, 45°, 90°, 135°), and the results were averaged to obtain stable descriptors. Given the cropped ROI resolution (104 × 224), small pixel offsets (d = 1–3) were selected to capture fine-grained texture variations within the joint region at a local scale while remaining robust to noise. The four standard orientations were used to account for directional texture patterns and reduce sensitivity to image orientation; averaging across distances and angles further improves descriptor stability. From these matrices, six classical statistical features were derived—contrast, dissimilarity, homogeneity, energy, correlation, and ASM—chosen as a compact, widely adopted set that summarizes complementary texture properties without excessively increasing the handcrafted feature dimensionality. These measures are well established in the literature and commonly available in standard image-processing libraries such as scikit-image. The computed GLCM vectors were generated for all images and stored as structured inputs, later fused with CNN-based features in the hybrid model to enhance classification performance.

### 3.5. Hybrid Model

**Figure 6:**
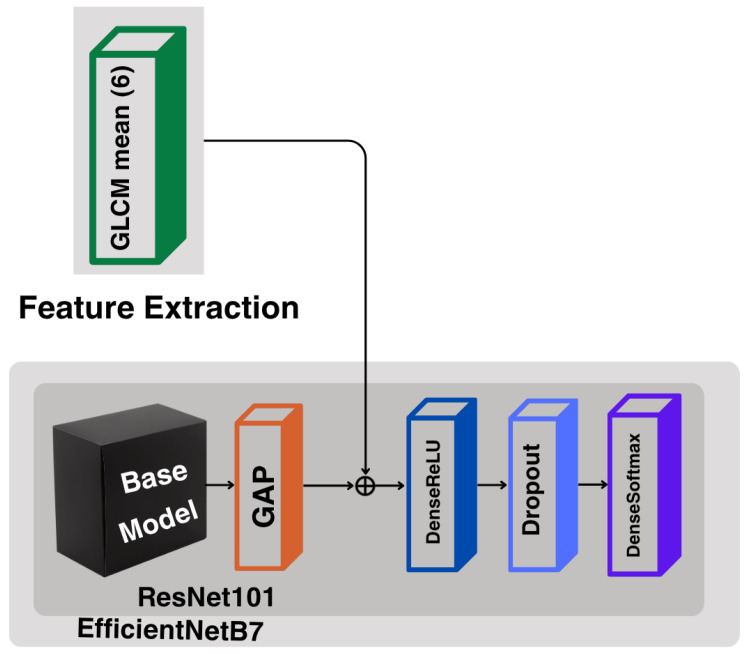
Hybrid model architecture combining CNN-based deep features and handcrafted GLCM descriptors.

To leverage both handcrafted and deep feature representations, a hybrid architecture was constructed by combining GLCM-derived features with deep features extracted from the two CNN backbones, ResNet-101 and EfficientNetB7. For each image, the handcrafted GLCM feature vector was computed in advance and kept constant during training, while the CNN-based features were optimized end-to-end. As shown in Figure 6, the two types of representations were concatenated after the global average pooling (GAP) layer, resulting in a unified feature vector that merges robust textural characteristics with high-level deep representations.

The fused feature vectors were subsequently passed through fully connected layers with ReLU activation and dropout regularization before the final softmax classification layer. This ensured that both handcrafted and deep features contributed equally to predicting the KL grade.

### 3.6. Experimental Setup

All experiments were executed on Google Colab Pro (colab.google), which provided sufficient computational resources to train the proposed hybrid model without major constraints. The implementation was developed in Python (Python.org) (v3.12.12) using TensorFlow (tensorflow.org) (v2.19.0) and Keras (keras.io) (v3.10.0). Both training and inference were run on an NVIDIA A100-SXM4 GPU (40 GB memory; NVIDIA Corporation, Santa Clara, CA, USA). The CUDA and cuDNN libraries were supplied by the Google Colab runtime environment employed throughout the experiments. To enhance reproducibility, random seeds were initialized for each execution.

### 3.7. Model Training

Each hybrid model was trained separately for the two backbones (ResNet-101 and EfficientNetB7) using the preprocessed and augmented dataset. Both CNNs were initialized with ImageNet weights and fine-tuned by unfreezing the last 20 layers, while earlier layers remained frozen. The training employed categorical cross-entropy as the loss function with Adam optimization, while the remaining hyperparameter settings are summarized in Table 2.

To mitigate overfitting, several regularization strategies were adopted: (i) dropout layers and L2 penalties within the classifier head, (ii) early stopping (https://www.tensorflow.org/api_docs/python/tf/keras/callbacks/EarlyStopping, accessed on 30 Jun 2025) (TensorFlow v2.16.1)) with patience monitoring of the validation loss, and (iii) a dynamic learning rate schedule (ReduceLROnPlateau) (https://www.tensorflow.org/api_docs/python/tf/keras/callbacks/ReduceLROnPlateau, accessed on 30 Jun 2025). In addition to oversampling, class weights were incorporated into the loss function to balance the contribution of underrepresented classes. Although both techniques were applied simultaneously, they address class imbalance at complementary stages: oversampling balances sample quantities during preprocessing, while class weights adjust the loss function based on the original class distribution. This combination, along with regularization techniques, resulted in stable training behavior and improved class-wise performance, particularly for minority classes (KL-3, KL-4). as will be further illustrated in the Results section.

### 3.8. Ensemble Strategy

**Figure 7:**
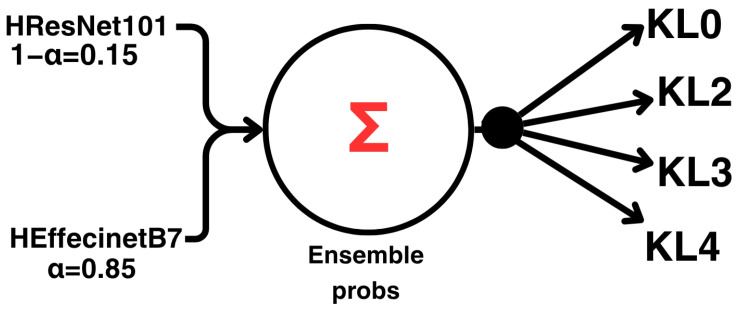
Ensemble strategy combining the outputs of the two hybrid models (ResNet-101 and EfficientNetB7).

As illustrated in Figure 7, the final prediction is obtained by computing a weighted average of the softmax output distributions from the two hybrid models, ResNet-101 and EfficientNetB7. The ensemble prediction is defined as:

(1) $$P_{\mathrm{ens}}=0.85\;P_{\mathrm{EfficientNetB}7}+0.15\;P_{\mathrm{ResNet}-101}$$

The larger weight assigned to EfficientNetB7 (α=0.85) reflects its better validation performance during model development, enabling the ensemble to capitalize on its stronger predictive capacity while still incorporating complementary information from ResNet-101. To determine these weights, we evaluated a small set of candidate combinations by varying the relative contributions of the two backbones, keeping all other training conditions fixed. The final weighting scheme was chosen based solely on validation-set metrics; the test set was not used in this process to avoid bias in the final evaluation. This weighted ensemble strategy enhances prediction robustness and generalization relative to using either model alone.

### 3.9. Performance Metrics

To assess the classification performance, four standard metrics were used: accuracy, precision, recall, and F1-score. These metrics are commonly applied in classification problems because they offer complementary perspectives on both overall correctness and class-level sensitivity. The formal mathematical definitions of these measures are presented in Table 3.

Alongside these quantitative metrics, Gradient-weighted Class Activation Mapping (Grad-CAM) was employed to improve model interpretability. This visualization method emphasizes the discriminative regions of the knee X-ray images that most strongly influenced the classification, thereby offering qualitative insight into the model’s decision-making process (Grad-CAM class activation visualization).

## 4. Results and Discussion

This section reports the experimental results obtained using the proposed method. The performance of the two hybrid models (ResNet-101 and EfficientNetB7) is first reported individually, followed by an ensemble scheme that aggregates their predictions. The models are assessed using accuracy, precision, recall, and F1-score, as defined in Section 3.8, and are further analyzed through confusion matrices and Grad-CAM heatmaps. Together, these results illuminate both the quantitative effectiveness and the interpretability of the proposed models.

### 4.1. Hybrid Models Performance

To establish a baseline, the two hybrid models—ResNet-101 and EfficientNetB7—were independently fine-tuned and evaluated on the test set. As summarized in Table 4, the EfficientNetB7 hybrid model obtained superior performance, achieving 72.2% accuracy, whereas the ResNet-101 hybrid model reached 68.1%. These findings motivated the adoption of an ensemble strategy to improve robustness and class balance further.

### 4.2. Ensemble Performance

As outlined in Section 3.7, the ensemble merged the outputs of the ResNet-101 and EfficientNetB7 hybrid models using a weighted averaging scheme. This ensemble achieved a test accuracy of 73%, surpassing the standalone models and exhibiting more balanced class performance. Table 5 presents the class-wise precision, recall, F1-score, and overall accuracy achieved by the ensemble model on the test set.

**Figure 8:**
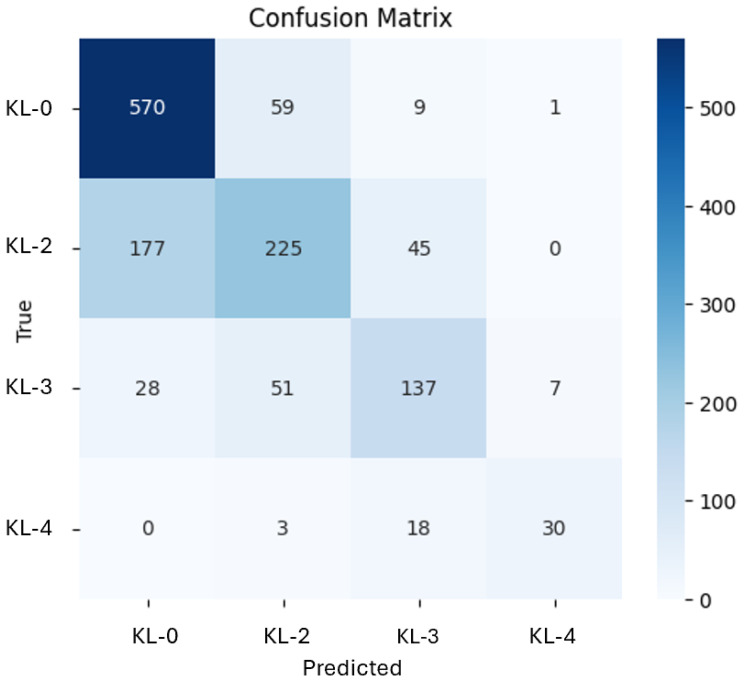
Confusion matrix of the ensemble model on the test set.

The confusion matrix in Figure 8 provides a detailed view of how predictions are distributed across the different KL grades. The ensemble reliably identifies normal cases (KL-0), whereas intermediate grades (KL-2, KL-3) are more difficult to classify, mirroring the inherent ambiguity of radiographic findings at these stages.

To further evaluate the robustness of the ensemble framework, we performed additional experiments under three distinct classification schemes: the full 5-class setting, a 3-class setting, and a binary setting. To account for statistical uncertainty arising from the finite test set size, we provide stratified bootstrap 95% confidence intervals [33,34] (2000 resamples) for the primary evaluation metrics in all configurations (Table 6). Overall, the resulting bootstrap CIs are relatively tight, suggesting that performance estimates on the held-out test set are stable. The 4-class configuration achieves higher accuracy and F1 scores than the 5-class setup, which aligns with the known ambiguity associated with KL-1.

When trained on the full Kellgren–Lawrence scale (0–4), the ensemble achieved 59% accuracy, indicating that correctly classifying the ambiguous KL-1 cases was challenging. This finding supports the decision to exclude KL-1 from the main 4-class setup, as label uncertainty at this level often leads to confusion between adjacent grades.

In the 3-class setup (KL-2, KL-3, KL-4), which included only degenerated cases, the ensemble achieved 74% accuracy. This suggests that the model becomes more stable and reliable at distinguishing between grades when trained exclusively on pathological categories, where radiographic differences are more apparent.

The ensemble obtained its best performance, 80% accuracy, when KL 0–1 were merged as “healthy” and KL ≥ 2 as “diseased,” demonstrating that it was highly effective at separating normal from osteoarthritic knees. The gradual improvement in performance across these configurations illustrates that the ensemble architecture adapts well to different levels of classification granularity, maintaining a good balance between generalization and clinical interpretability.

**Figure 9:**
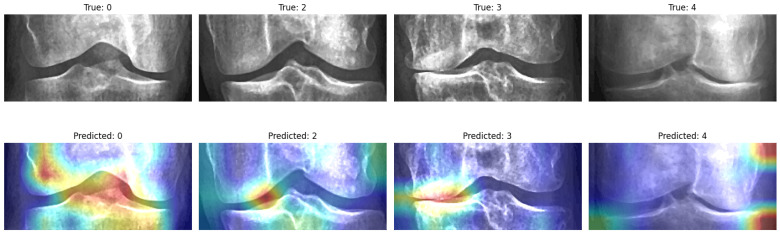
Grad-CAM visualizations highlighting discriminative regions across different KL grades. The top row shows correctly classified cases with attention focused on clinically relevant joint space and osteophyte regions. The bottom row presents representative misclassifications, in which attention patterns reveal ambiguous intermediate features that contribute to prediction errors between adjacent grades. In the heatmaps, warmer colors (red/yellow) indicate higher model attention, whereas cooler colors (blue) denote lower contribution to the prediction.

In addition to the quantitative analysis, Grad-CAM was applied directly to the ensemble model, rather than to an individual hybrid backbone, to visualize the image regions that most strongly influenced the final predictions. As shown in Figure 9, the resulting maps offer class-specific interpretations:

- KL-0 (normal cases): the model’s attention is spread over the entire joint structure, consistent with the lack of localized degeneration patterns.
- KL-2 and KL-3 (mild to moderate degeneration): the ensemble focuses more narrowly on the joint-space intersection, which corresponds to the gap narrowing that typically defines these grades.
- KL-4 (severe cases): the attention maps appear more diffuse, highlighting multiple regions of degradation, in line with the extensive structural damage characteristic of this stage.

Collectively, these observations demonstrate that the ensemble-based Grad-CAM not only emphasizes clinically meaningful areas but also adjusts its focus according to disease severity, thereby supporting both the predictive performance and the interpretability of the proposed approach.

### 4.3. Reducing Overfitting

**Figure 10:**
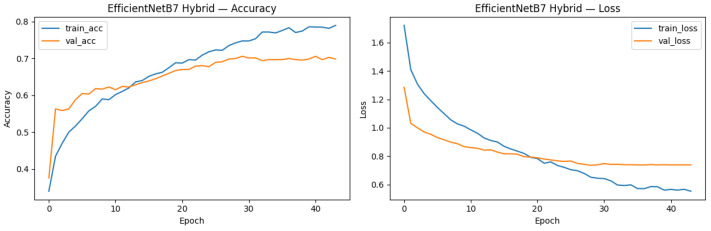
Training and validation curves before hyperparameter adjustments.

The influence of hyperparameter optimization and class weighting was clearly reflected in the training behavior. As illustrated in Figure 10, the baseline EfficientNetB7 model without further tuning exhibited clear overfitting: training accuracy continued to rise, whereas validation accuracy quickly reached a plateau, and the training and validation loss curves progressively diverged.

In contrast, the hybrid model trained with optimized hyperparameters (including dropout, L2 regularization, and learning rate scheduling) and balanced class weights exhibited more stable learning curves (Figure 10). Training and validation accuracy improved in parallel, and the validation loss consistently declined before leveling off, suggesting enhanced generalization and reduced overfitting.

Overall, these observations demonstrate that the applied regularization techniques and class re-weighting effectively mitigated class imbalance and increased the robustness of the training process.

### 4.4. Ablation Study

Table 7 indicates that each component yields a positive contribution, with the most substantial improvement arising from the removal of KL-1, which is affected by label ambiguity. The subsequent additions (class-weighted loss, GLCM fusion, and ensembling) offer further gains, culminating in the best overall performance in the final setup.

To enhance the robustness of our analysis, we additionally performed a component-wise ablation by independently toggling (i) handcrafted GLCM features and (ii) the ensemble strategy. Table 8 reports the separate and combined impacts of these two elements. Adding GLCM features boosts the performance of both backbone models, and the ensemble strategy further increases robustness. The highest performance is obtained when both components are used together.

### 4.5. Comparison with Baseline

Given that few studies have explored alternative grading schemes for knee osteoarthritis (KOA) using comparable methodologies, our analysis is centered on the most relevant baseline, DL + XAI [27]. That work used the full five-level Kellgren–Lawrence (KL) scale (0–4), achieving accuracies of 0.56 for multi-class classification and up to 0.76 for binary discrimination.

By contrast, our study proposes a revised class configuration that deliberately omits the uncertain KL-1 grade, thereby reducing ambiguity in intermediate cases while preserving clinically meaningful separations. We further improved the image preprocessing pipeline with a dedicated knee-joint-centered approach and reinforced the overall framework by incorporating handcrafted GLCM texture descriptors and applying a weighted ensemble of EfficientNetB7 and ResNet-101 to exploit their complementary strengths. Collectively, these choices ensure that the radiographs consistently highlight the clinically relevant joint region and minimize background variability. Under the full five-class KL setting (0–4), this dedicated preprocessing leads to better performance, achieving an accuracy of **0.59** compared with the DL + XAI baseline of 0.56 [27]; likewise, for the analogous binary setup (0 + 1) vs. (2 + 3 + 4), our method attains **0.80** versus 0.68 [27]. Since KL-1 is intrinsically ambiguous—often reflecting very subtle or equivocal radiographic changes—it can introduce label noise and reduce consistency in intermediate cases. Consequently, we also investigate an uncertainty-aware four-class configuration (0, 2, 3, 4) to evaluate performance when this borderline grade is removed. This adjustment yields a four-class scheme (0, 2, 3, 4) that improves label reliability without overly simplifying the problem. Overall, these findings indicate that explicitly managing label uncertainty can provide performance gains even in the absence of multimodal inputs or transformer-based architectures. The comparison with existing baseline studies is presented in Table 9. Additional experimental results, including confusion matrices for the 5-class, 3-class, and binary configurations, are provided in Appendix A.

## 5. Conclusions

In this study, we introduced a hybrid ensemble framework for automatic grading of knee osteoarthritis (KOA) from X-ray images. The method combines handcrafted texture descriptors derived from the Gray Level Co-occurrence Matrix (GLCM) with deep feature representations extracted from pre-trained CNN models. By integrating hybrid models based on ResNet-101 and EfficientNetB7 in a weighted ensemble, the framework effectively leveraged the complementary capabilities of both backbones.

Experimental results showed that the ensemble approach achieved higher accuracy (73%) than the individual hybrid models, indicating greater robustness and generalization. Grad-CAM visualizations additionally revealed that the model predominantly attends to clinically meaningful joint structures, thereby supporting its interpretability.

In summary, this work underscores the advantages of combining handcrafted features with deep representations, as well as the effectiveness of ensemble learning for reliable KOA severity assessment. Future research may focus on expanding the dataset, improving feature fusion mechanisms, and adopting more advanced architectures to further enhance diagnostic precision and clinical utility. 

## Figures and Tables

**Table 1:** Data augmentation techniques and their corresponding parameter settings were used to enhance the training dataset.

Augmentation Technique	Value
Rotation	±18°
Flip horizontally only	
Brightness adjustment	±15%
Contrast adjustment	±15%
Translation	5% (height & width)
Gaussian noise	0.02

**Table 2:** Training hyper-parameters.

Parameter	Name/Value
Optimizer	Adam
Loss function	Categorical cross-entropy
Batch size	32
Epochs	100
Learning rate	$1\times 10^{-5}$
Early stopping	Patience = 15
LR scheduler	ReduceLROnPlateau (factor = 0.5, patience = 3, min_lr = $1\times 10^{-7}$)
Class weights	Applied
Regularization	Dropout (0.6, 0.3), L2 = $1\times 10^{-4}$

**Table 3:** Performance metrics are used for evaluation.

Metric	Formula	Description
Accuracy	$\frac{TP+TN}{TP+TN+FP+FN}$	Overall proportion of correct predictions
Precision	$\frac{TP}{TP+FP}$	Correctly predicted positives among all predicted positives
Recall	$\frac{TP}{TP+FN}$	Correctly predicted positives among all actual positives (sensitivity)
F1-score	$2\times \frac{\mathrm{Precision}\times \mathrm{Recall}}{\mathrm{Precision}+\mathrm{Recall}}$	Harmonic mean of precision and recall

**Table 4:** Test the accuracy of individual hybrid models.

Model	Test Accuracy
ResNet-101 Hybrid	0.681
EfficientNetB7 Hybrid	0.722

**Table 5:** Classification performance of the ensemble model on the test set.

Class/Metric	Precision	Recall	F1-Score
KL-0	0.78	0.87	0.83
KL-2	0.65	0.60	0.62
KL-3	0.72	0.60	0.65
KL-4	0.80	0.63	0.70
Accuracy			0.73
Macro Avg	0.74	0.68	0.70
Weighted Avg	0.73	0.73	0.73

**Table 6:** Bootstrap 95% confidence intervals (CIs) for the proposed ensemble framework across multiple KOA classification settings (2000 stratified bootstrap resamples on the held-out test set).

Setting	KL Classes	Accuracy	Macro-F1	Weighted-F1/F1
Multi-class (4)	(0, 2, 3, 4)	0.722 [0.699–0.744]	0.684 [0.644–0.719]	0.720 [0.696–0.742]
Multi-class (5)	(0, 1, 2, 3, 4)	0.585 [0.563–0.606]	0.570 [0.539–0.598]	0.569 [0.547–0.592]
Multi-class (3)	(2, 3, 4)	0.770 [0.741–0.798]	0.702 [0.656–0.745]	0.761 [0.729–0.790]
Binary	(0 + 1) vs. (2 + 3 + 4)	0.795 [0.777–0.814]	–	0.731 [0.704–0.759]

**Table 7:** Incremental ablation results of the proposed framework in the 4-class setting (KL-0, 2, 3, 4). The best-performing configuration is highlighted in bold.

Step	Configuration	Test Accuracy
A0	Preprocessing	0.56
A1	A0 + augmentation	0.57
A2	A1 + exclude KL-1 (4-class)	0.69
A3	A2 + class-weighted loss	0.70
A4	A3 + GLCM fusion	0.72
A5	A4 + ensemble	**0.73**

**Table 8:** Component-wise ablation in the 4-class setting (KL-0, 2, 3, 4): individual and combined effects of GLCM features and ensemble learning on classification accuracy. 🗸 indicates inclusion of the component, while × denotes its exclusion. The best performance is highlighted in bold.

GLCM	Ensemble	ResNet-101 Acc	EfficientNetB7 Acc	Ensemble Acc
×	×	0.61	0.70	–
🗸	×	0.65	0.72	–
×	🗸	0.62	0.70	0.70
🗸	🗸	0.68	0.72	**0.73**

**Table 9:** Comparison with Baseline studies. The best-performing configuration is highlighted in bold.

Study	Classification Type	Classes Considered	Reported Accuracy
DL + XAI [27]	Multi-class (5)	(0, 1, 2, 3, 4)	0.56
	Binary	(0 + 1) vs. (2 + 3 + 4)	0.68
	Binary	(0–2) vs. (3–4)	0.76
Proposed Method	Multi-class (5)	(0, 1, 2, 3, 4)	**0.59**
	Multi-class (4)	(0, 2, 3, 4)	**0.73**
	Multi-class (3)	(2, 3, 4)	0.74
	Binary	(0 + 1) vs. (2 + 3 + 4)	**0.80**

**Figure A1:**
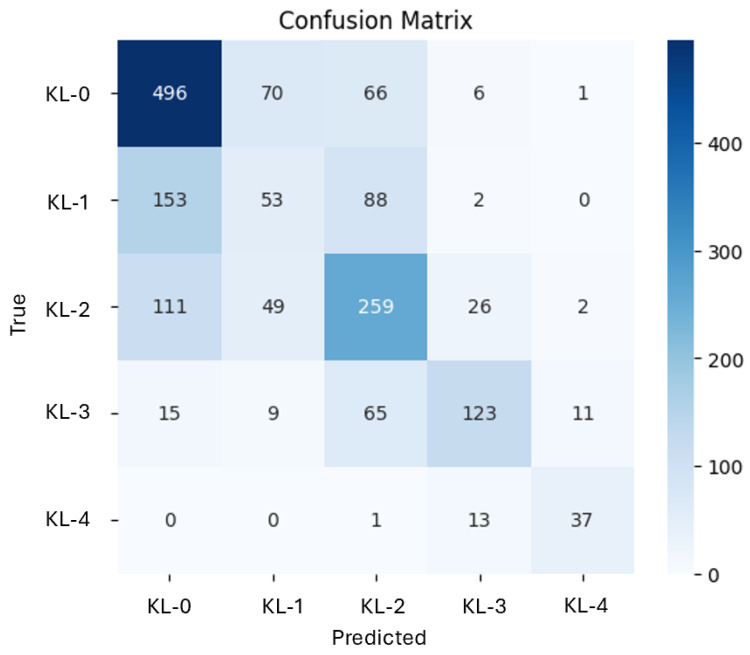
Confusion matrix of the ensemble model for 5-class configuration.

**Figure A2:**
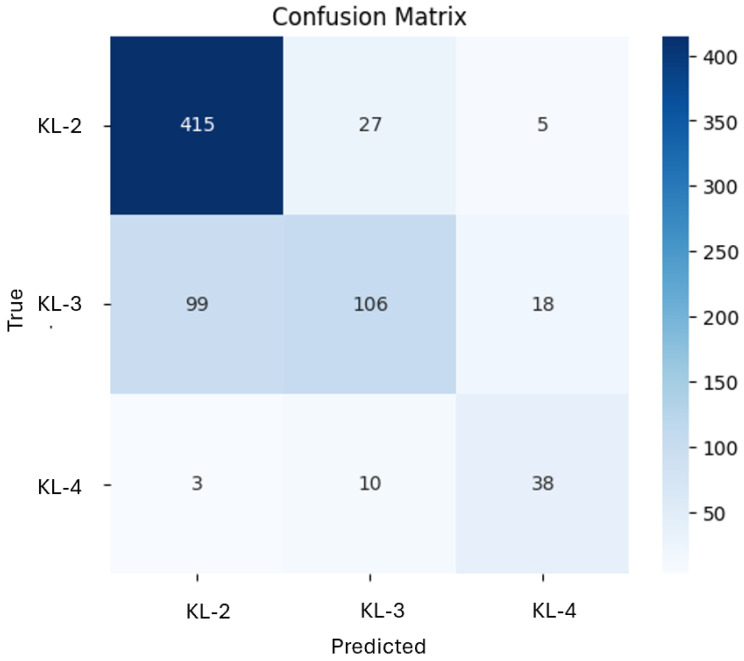
Confusion matrix of the ensemble model for 3-class configuration.

**Figure A3:**
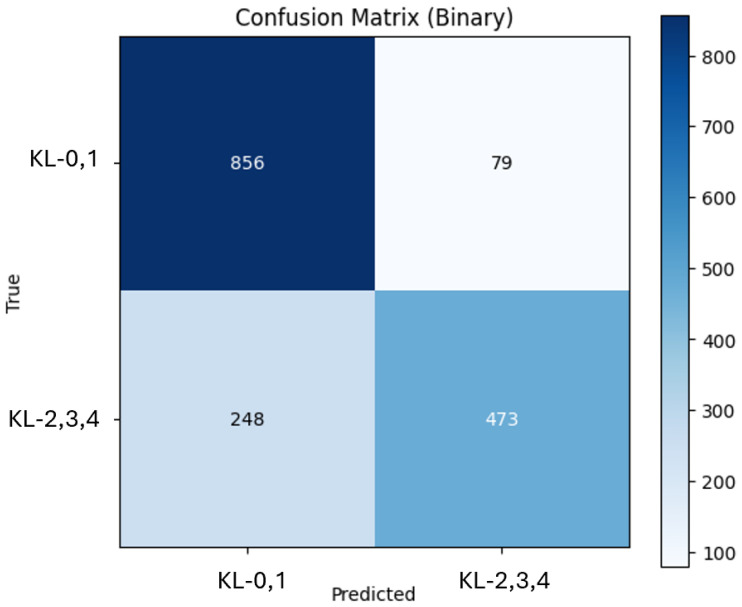
Confusion matrix of the ensemble model for binary configuration.

## Data Availability

The dataset used in this study is publicly available at Mendeley Data: https://data.mendeley.com/datasets/56rmx5bjcr/1, accessed on 30 June 2025 (DOI: https://doi.org/10.17632/56rmx5bjcr.1).
